# Advances in Glaucoma Diagnosis and Treatment: Integrating Innovations for Enhanced Patient Outcomes

**DOI:** 10.3390/biomedicines13040850

**Published:** 2025-04-02

**Authors:** Shih-Heng Hung, Wei-Ting Yen, Da-Wen Lu

**Affiliations:** 1 Department of General Medicine, Tri-Service General Hospital, National Defense Medical Center, Taipei 11490, Taiwan; a91236a91236@gmail.com; 2 Department of Ophthalmology, Tri-Service General Hospital, National Defense Medical Center, Taipei 11490, Taiwan

Glaucoma, a chronic and progressive eye disease, is a significant public health concern responsible for a substantial proportion of global vision impairment and blindness [1]. The diagnosis and treatment of glaucoma have been challenging, with early detection critical to mitigate the risk of irreversible vision loss [2]. Trabeculectomy and pharmacological management have been the mainstay of treatment, but novel approaches are emerging that aim to improve outcomes and reduce the burden of this disease. Currently, artificial intelligence-based tools are being explored to enhance glaucoma detection [3], monitoring [4], and prediction of treatment response [5]. In this Special Issue, we discussed the latest advancements in the field of glaucoma, focusing on new diagnostic and therapeutic approaches that have the potential to transform patient care.

## 1. Novel Diagnostic Techniques

- Diverse Diagnostic Technologies

The early identification of glaucoma is crucial, as it allows for timely intervention and management to preserve visual function [6]. While intraocular pressure (IOP) measurement remains a fundamental aspect of the evaluation, contact lens-based sensors have recently emerged as a promising alternative, enabling continuous monitoring [7]. Intraocular telemetric IOP sensor systems demonstrate significant potential to provide real-time IOP data, aiding in the identification of diurnal IOP fluctuations and facilitating the development of personalized treatment regimens [8]. The emergence of these technologies signifies a major advancement in glaucoma diagnosis, transitioning from single-point measurements to comprehensive monitoring, laying the foundation for more precise disease management.

For instance, recent studies have explored continuous IOP monitoring using contact lens sensors. Gillmann et al. (2021) and Wasilewicz et al. (2020) tested a pressure-measuring contact lens (PMCL) that provided 24-h IOP measurements in mmHg. Both studies found good comparability with standard tonometry, with 88% and 97.2% of measurements within 5 mmHg of pneumatonometry and dynamic contour tonometry, respectively [9,10]. Zhang et al. (2024) discovered that normal Chinese adults exhibit stable 24-h intraocular pressure patterns, with comparable mean levels during the day and night, as well as scattered peak and trough times [8]. These non-invasive devices can detect subtle IOP variations, such as those induced by water-drinking tests [9,10]. While promising, some studies noted potential corneal complications [9], indicating the need for further research and refinement. These studies demonstrate that contact lens sensors hold potential for clinical application, but challenges related to safety and long-term stability must be addressed to fully realize their utility.

Furthermore, advancements in diagnostic technologies are not limited to imaging and intraocular pressure monitoring but also extend to systemic evaluations. Iannucci et al. (2024) (contribution 1) investigated the potential association between olfactory dysfunction and glaucoma. They found that patients with exfoliation glaucoma (PXG) exhibited significantly reduced olfactory identification and sensitivity, which may be related to protein aggregation abnormalities and impaired vascular regulation. In the Sniffin’ Stick Test (SST), PXG patients showed greater olfactory impairment compared to those with primary open-angle glaucoma (POAG), suggesting that olfactory dysfunction could serve as a potential early biomarker for glaucoma. (contribution 1) Olfactory testing provides a non-invasive and rapid screening method, making it particularly suitable for high-risk populations, such as individuals with exfoliation syndrome. However, to establish its clinical utility as a diagnostic adjunct, a cross-sectional diagnostic study is needed to accurately determine its sensitivity and specificity, particularly in resource-limited settings.

- Advancements in Structural Assessment Technologies

A recent study (contribution 2) using a steroid-induced glaucoma (SIG) model demonstrated that optical coherence tomography (OCT) can assess retinal structure and analyze the size and shape of vitreous hyperreflective opacities to distinguish immune cell activation states. The study found a strong correlation between these immune cell changes and elevated intraocular pressure and tissue damage over time. Given these findings, this imaging-based approach may serve as a novel biomarker for monitoring immune activation and disease progression in glaucoma, providing a non-invasive tool for early detection and therapeutic evaluation.

Studies have demonstrated that wide-field and peripapillary retinal nerve fiber layer (RNFL) thickness measurements using swept-source OCT (SS-OCT) exhibit comparable diagnostic accuracy to spectral-domain OCT (SD-OCT) in detecting glaucomatous damage [11]. Kim et al. (2020) demonstrated that SS-OCT wide-field maps provide better accuracy for detecting glaucomatous defects and exhibit greater diagnostic power in myopic eyes compared to SD-OCT [12]. Tsai et al. (2024) (contribution 3) highlighted the importance of structural biomarkers in glaucoma diagnosis, emphasizing that SS-OCT provides superior imaging depth compared to SD-OCT, allowing for the clearer visualization of deep optic nerve structures such as the lamina cribrosa and scleral spur. SS-OCT enables the precise measurement of scleral spur length (SSL), which is essential for assessing anterior chamber angle structures and aqueous outflow pathways, thereby aiding in identifying high-risk glaucoma patients. Furthermore, the lamina cribrosa morphology, including posterior displacement, deformation, and thinning, plays a critical role in optic nerve damage, and SS-OCT offers a more detailed evaluation of these changes compared to SD-OCT (contribution 3).

Ultrasound biomicroscopy (UBM) is essential for diagnosing and planning surgery in primary congenital glaucoma (PCG) [13]. Janssens et al. (2022) indicated that UBM effectively visualizes structural abnormalities in PCG, such as an enlarged trabecular–iris angle, reduced iris thickness, and Schlemm’s canal narrowing or absence. Additionally, UBM aids in assessing disease severity and identifying anatomical obstacles that may impact surgical planning [13]. Another study also concluded that thinner iris thickness and larger pupil diameter are risk factors for surgical failure, highlighting the clinical value of UBM in guiding treatment strategies [14]. Moreover, both anterior segment optical coherence tomography (AS-OCT) and UBM are useful for anterior segment assessment, particularly in measuring anterior chamber depth (ACD) [15]. A meta-analysis found no statistically significant difference in ACD measurements between the two modalities (SMD = 0.19, 95% CI [0.00, 0.39]); however, further high-quality studies are needed due to limited sample sizes [15]. In clinical practice, AS-OCT offers high-resolution and rapid imaging, making it more suitable for routine examinations. However, for uncooperative patients, such as young children, UBM is preferable as it can be performed under general anesthesia [16].

Wang et al. (2019) emphasized that AS-OCT, with its high resolution and fast imaging capabilities, has become a key tool for diagnosing both glaucoma and corneal diseases. By measuring anterior chamber angle parameters (e.g., the angle-opening distance and angle recess area), AS-OCT is especially useful for diagnosing and monitoring angle-closure glaucoma [17]. Desmond et al. (2022) conducted a systematic review and meta-analysis comparing AS-OCT with gonioscopy in detecting angle closure, showing that AS-OCT reached sensitivities and specificities of 87% and 84%, respectively. However, its limited field of view and relatively high false-positive rate make it more suitable as a screening tool [18]. Incorporating deep learning (DL) pushed sensitivity to 94% and specificity to 93.6%, highlighting the feasibility of AI-assisted image analysis—though more long-term clinical research is required to confirm its value [19]. These novel diagnostic technologies not only broaden the scope of early screening but also underscore the need to utilize DL or machine learning (ML) to improve diagnostic accuracy. This will be a key challenge to address in future clinical practice.

## 2. Advanced Therapeutic Interventions

- Emerging Drug Therapies

1. Sustained-Release Systems

Several Phase III clinical trials have assessed various sustained-release implants. Medeiros et al. (2020) showed that bimatoprost implants (10 μg and 15 μg) were noninferior to timolol in IOP reduction. However, the 15 μg implant had a higher incidence of corneal endothelial cell loss and inflammation, making the 10 μg dose the safer option. Endothelial cell loss may result from direct contact between the implant and the cornea, with polymer matrix accumulation in the angle potentially exacerbating the damage. Given this risk, repeated administration should be approached with caution to prevent further endothelial compromise [20].

Bacharach et al. (2021) further reported that, after the third administration at week 32, 77.5% of patients receiving the 10 μg bimatoprost implant required no additional treatment for one year, with slower visual field progression compared to the timolol-treated group [21]. In addition, travoprost implants (fast-eluting [FE] and slow-eluting [SE] types) demonstrated IOP-lowering efficacy comparable to timolol at three months, with good overall safety profiles. However, one serious adverse event (endophthalmitis) was reported in the SE implant group, highlighting a potential risk associated with the injection procedure [22]. In 2020, the FDA approved DURYSTA, a bimatoprost sustained-release (BimSR) intracameral implant. However, its primary safety concern is corneal endothelial cell loss (ECL), which worsens with repeated use [23]. As a result, DURYSTA is restricted to a single implant per eye, with no repeat treatments allowed. While its main advantage lies in improving patient adherence, the issue of ECL remains unresolved.

Both 10 μg and 15 μg bimatoprost implants, as well as travoprost implants, demonstrated IOP-lowering efficacy comparable to traditional medications such as timolol. These findings confirm the potential of sustained-release medications to reduce treatment burden, improve patient adherence, and slow visual field progression, offering a more flexible approach to the long-term management of glaucoma. However, further research on long-term safety is necessary to ensure feasibility and reliability in widespread application.

2. Rho Kinase Inhibitors (RKIs)

RKIs are a class of emerging anti-glaucoma drugs that effectively lower IOP by enhancing trabecular meshwork (TM) outflow and reducing extracellular matrix (ECM) stiffness. Liu et al. (2024) reported that ripasudil and netarsudil have been approved for use in Japan and the United States, respectively [24]. However, their IOP-lowering efficacy appears to be less pronounced than timolol, as Nana et al. (2024) found that RKIs achieved slightly lower short-term IOP reduction, though this difference was not clinically significant [25]. Despite this, RKIs may offer additional benefits, including potential neuroprotection by improving optic nerve blood flow and reducing retinal ganglion cell apoptosis, as well as anti-fibrotic effects that could help minimize postoperative scarring and enhance surgical success rates. These secondary effects warrant further investigation to determine their long-term clinical value [24]. In terms of safety, RKIs were associated with a higher incidence of localized side effects, such as conjunctival hyperemia, which were mostly mild and reversible [24,25].

To address these concerns, a novel Rho-associated protein kinase 2 (ROCK2)-selective inhibitor, ITRI-E-212, has demonstrated comparable IOP-lowering efficacy while significantly reducing conjunctival hyperemia. In animal studies, 1% ITRI-E-212 lowered IOP by 24.9% in normotensive rabbits and 28.6% in ocular hypertensive rabbits, with effects lasting at least six hours. Importantly, only transient and mild hyperemia was observed, which was resolved within six hours, marking a significant advantage over existing RKIs [26].

Therefore, RKIs provide a viable alternative to traditional therapies, offering comparable IOP-lowering efficacy with a more favorable systemic safety profile. While their short-term IOP reduction may be slightly less pronounced than timolol, RKIs remain a safer option, particularly for patients who cannot tolerate β-blockers due to cardiovascular or respiratory concerns. Additionally, the development of ITRI-E-212, a ROCK2-selective inhibitor with reduced conjunctival hyperemia, helps overcome a key limitation of existing RKIs and may improve patient adherence. Further research is needed to validate the long-term safety of RKIs and to explore their broader neuroprotective and anti-fibrotic benefits in glaucoma management.

3. Advances in Neuroprotective Therapies

The core pathology of glaucoma involves the progressive degeneration of retinal ganglion cells (RGCs) and optic nerve damage [27]. While current treatments primarily focus on lowering IOP, many glaucoma patients still experience vision loss due to ongoing neurodegeneration [28]. This indicates that simply lowering IOP cannot completely halt the progression of neurodegeneration. Consequently, neuroprotective therapies are gradually becoming a focal point in glaucoma research and clinical applications.

A review on glaucoma treatment highlighted several emerging neuroprotective therapies, such as neurotrophic factors (NTFs), citicoline, and nicotinamide. Among these, the ciliary neurotrophic factor (CNTF) has become a focal point in glaucoma neuroprotection research due to its ability to promote neuronal survival, repair, and regeneration [29]. A Phase I clinical trial by Goldberg et al. further supported this perspective [30]. The trial involved 11 patients with POAG, with one eye receiving the CNTF-secreting NT-501 implant while the other eye served as a control. Over an 18-month follow-up, NT-501 demonstrated good safety, with only mild postoperative foreign body sensations that were resolved within 12 weeks. Importantly, the implanted eyes showed positive outcomes in both function and structure, including stabilized visual acuity, slight improvements in contrast sensitivity, and a significant increase in RNFL thickness. In contrast, the control eyes exhibited functional decline [30]. This study underscores CNTF’s potential in neuroprotection and neuroenhancement, paving the way for novel glaucoma treatments. Another NTF under investigation is the recombinant human nerve growth factor (rhNGF). Animal studies have shown that rhNGF activates the TrkA receptor and PI3K/Akt signaling pathway, reducing apoptosis and promoting neuronal regeneration, thereby protecting RGCs [31]. A study evaluating the use of 180 μg/mL rhNGF eye drops in patients with open-angle glaucoma (OAG) included 60 participants who underwent 8 weeks of treatment followed by a 24-week follow-up. The results demonstrate that rhNGF had good safety and tolerability, with no treatment-related serious adverse events. However, in the short term, it did not achieve significant neuroenhancement effects. Nevertheless, functional and structural measures showed a non-significant trend favoring rhNGF, suggesting its potential neuroprotective role [32].

Another potential drug, citicoline, has been widely studied for its neuroprotective effects in glaucoma. Numerous studies have shown that citicoline can reduce RGC loss and improve visual field performance, VEPs, and PERG while preserving RNFL and ganglion cell complex (GCC) thickness [33,34,35]. Currently, a one-year randomized controlled trial (RCT) (NCT05315206) is evaluating the effects of oral citicoline in patients with OAG, and a large Phase III RCT (NCT05710198) is planned to further investigate whether citicoline eye drops can slow visual field deterioration and structural changes in glaucoma. These studies will provide key evidence to assess the clinical value of citicoline in glaucoma treatment. In addition, Babighian et al. (2024) (contribution 4) explored the potential of nicotinamide (vitamin B3) to increase nicotinamide adenine dinucleotide (NAD+) levels, reduce oxidative stress, and protect RGCs.

These findings suggest that neuroprotective drug therapies are revolutionizing glaucoma management, offering promising directions for future treatment strategies.

- Progress in Novel Surgical Techniques

In addition to pharmacological therapies, advancements in surgical techniques have also expanded the options available for glaucoma treatment. In the surgical domain, Bolek et al. (2024) (contribution 5) evaluated the five-year outcomes of endoscopic cyclophotocoagulation (ECP) combined with phacoemulsification. Their results showed an average IOP reduction of approximately 32.7%, with a low risk of complications, though the long-term success rate was limited; 40.6% of patients required continued medication at 60 months post-surgery.

A study conducted by Bolek et al. (2023) (contribution 6) also demonstrated the efficacy of Ahmed valve implantation (AV) in patients with aniridic glaucoma. Postoperative IOP showed an average reduction of 40.2%, with a qualified success rate of 85.7%. No severe complications were observed, and only mild-to-moderate subconjunctival hemorrhage was noted. These findings suggest that both traditional and novel surgical approaches offer distinct advantages in glaucoma management, and the choice of procedure should be tailored to the patient’s specific characteristics.

Furthermore, Preserflo MicroShunt surgery, as a minimally invasive glaucoma surgery (MIGS), has been proven effective in lowering IOP. Storp et al. (2023) (contribution 7) reported that postoperatively, the median IOP decreased by 8 mmHg, with minimal impact on macular flow density (FD). However, a significant reduction in peripapillary radial capillary (RPC) FD was observed, particularly in advanced glaucoma patients. This observation highlights the importance of assessing RPC FD before surgery to evaluate the suitability of Preserflo implantation. For patients with an already reduced RPC FD, careful consideration is necessary to mitigate the potential risk of further decline [36]. (contribution 7).

We believe these results mark a significant shift in glaucoma management, as maintaining optic nerve function and stabilizing visual field progression are no longer solely dependent on IOP-lowering mechanisms. Both pharmacological and surgical interventions are advancing toward “neurostructural protection” and “long-term therapeutic efficacy”. Given these varied options, clinical practice should focus on personalized strategies, matching treatments to each patient’s disease progression and other health conditions.

## 3. Artificial Intelligence and Digital Health

Artificial intelligence (AI) has demonstrated unprecedented potential in diagnosis, the prediction of disease progression, and surgical evaluation of glaucoma. With advancements in technology, AI enhances clinical decision-making by providing more accurate analyses, optimizing treatment strategies, and supporting personalized patient management. Its ability to integrate multimodal data and detect subtle disease patterns makes it a valuable tool for improving outcomes in glaucoma care.

- Diagnosis of Glaucoma

Phene et al. (2019) developed a deep learning model for analyzing fundus photographs, training it on a large dataset, and validating its performance across different cohorts. The model effectively detected referable glaucomatous optic neuropathy (GON) by identifying key features such as an increased cup-to-disk ratio, RNFL defects, and neuroretinal rim notching. Notably, its performance surpassed that of multiple glaucoma specialists. This AI model holds promises for glaucoma screening and diagnostic support, particularly in resource-limited settings, where it may enhance early detection and reduce missed diagnoses [37]. Meanwhile, Chiang et al. (2024) employed wide-field OCT to simultaneously capture three-dimensional structures of both the macula and optic nerve head, reaching an AUC of 0.99 [38]. In the domain of diagnosing glaucoma in high myopia, another study (contribution 8) adopted a ConvNeXt_Base model combined with a convolutional block attention module (CBAM), achieving an AUC of 0.894—demonstrating the feasibility of large-scale, rapid screening through deep learning. However, the study also emphasized the need to incorporate more diverse datasets, such as OCT and visual field test results, to further improve the model’s generalizability. To address the challenge of AI diagnostic performance requiring large datasets, recent studies (contribution 9) have demonstrated that Few-Shot Learning (FSL) combined with wide-field optical coherence tomography angiography (WF-OCTA) can significantly enhance diagnostic accuracy even in data-scarce scenarios. For instance, using data from only 195 eyes, the AUC reached 0.93, outperforming traditional deep learning methods with an AUC of 0.80. This highlights the potential of AI technologies to advance novel imaging diagnostics.

- Prediction of Disease Progression

Beyond diagnosis, AI has also gained attention in predicting glaucoma progression. Mohammadzadeh et al. (2024a) demonstrated that integrating RNFL thickness with a series of three optic disk photographs (ODPs) using ResNet-152 improved the AUC to 0.894 for predicting visual field progression [39]. In another study, Mohammadzadeh et al. (2024b) employed ResNet-50 to analyze the baseline and the most recent ODP, achieving an AUC of 0.926 with excellent sensitivity and specificity [40]. Moreover, genetic screening has also been integrated into AI applications (contribution 10). For instance, incorporating specific single-nucleotide polymorphisms (SNPs) of the LOXL1 gene, such as rs2165241 and rs1048661, into risk models for exfoliation glaucoma (PXG) improved the model’s AUC by 10–15%, increasing from 0.75 to 0.85–0.90. This demonstrates that combining genetic testing with AI can enable more precise personalized treatment strategies. Additionally, another study (contribution 11) using Mendelian randomization analysis identified a significant causal relationship between type 2 diabetes (T2D), fasting glucose (FG) levels, and the risk of POAG. T2D was found to increase POAG risk (OR = 1.06), with FG having an even stronger impact (OR = 1.19). These results suggest that metabolic dysregulation may contribute to glaucoma progression. Given the increasing role of AI in disease prediction, incorporating systemic and genetic risk factors identified in genome-wide association study (GWAS) and Mendelian randomization (MR) studies could enhance the accuracy of AI-based glaucoma progression models (contribution 11).

- Evaluation of Surgical Outcomes

AI demonstrates significant potential in evaluating surgical outcomes for glaucoma. Birla et al. (2024) focused on trabeculectomy for Juvenile Open-Angle Glaucoma (JOAG), analyzing preoperative and intraoperative parameters through ML models. They identified age, preoperative IOP, the thickness of Tenon’s capsule, the use of mitomycin C (MMC), and surgical techniques as the main factors affecting five-year postoperative success rates. Using an IOP reduction ≥ 50% as the success criterion, the study showed that three different algorithmic models achieved accuracy rates of 86.45%, 87.47%, and 87.44%, with average AUC values of 0.876, 0.926, and 0.922, demonstrating excellent performance in distinguishing successful from failed cases [41].

On the other hand, Banna et al. (2022) evaluated the performance of different ML models, including random forest, support vector machine, artificial neural network, and logistic regression, in predicting one-year postoperative success rates for refractory glaucoma patients. The results showed that random forest performed best, with an accuracy of 68% and an AUC of 0.74. The study further found that systemic health factors, such as the history of myocardial infarction, may influence the outcomes of trabeculectomy [42].

Barry (2024) utilized electronic health record (EHR) data to develop various models predicting outcomes for multiple glaucoma surgeries, including trabeculectomy, MIGS, and drainage device implantation. The study included 2398 surgical cases and showed that the random forest model performed best, with an accuracy of 75.5% and an AUC of 76.7%. Notably, the model demonstrated a higher predictive ability for postoperative IOP control (AUC = 86%) but a relatively lower ability to predict surgical failure or increased postoperative medication requirements, indicating that different prediction targets may require different AI models to optimize the results [5].

Integrating multidimensional data is crucial for improving AI model performance, especially when accounting for diverse patient characteristics, such as systemic health factors. AI has shown strong potential in optimizing surgical success rates and reducing postoperative complications, offering a pathway toward personalized patient management. However, challenges such as data standardization, privacy concerns, and the complexity of multimodal data processing remain barriers in clinical practice. Addressing these issues is essential for fully realizing AI’s potential in glaucoma care.

## 4. Conclusions

Recent advancements in research and clinical practice indicate that glaucoma diagnosis and treatment are becoming increasingly integrated and precise. A multidimensional approach enables earlier detection, personalized disease monitoring, and targeted therapeutic strategies.

In diagnostics, continuous IOP monitoring, olfactory function testing, AS-OCT, and UBM offer valuable tools for identifying high-risk patients, optimizing surgical planning, and improving postoperative evaluations.

In pharmacological treatment, Rho kinase inhibitors, neurotrophic factors such as CNTF and rhNGF, and emerging metabolism-targeting therapies like nicotinamide and citicoline provide new possibilities for managing glaucoma progression.

In surgical innovations, advancements such as ECP combined with phacoemulsification, Ahmed valve implantation (AV), and Preserflo MicroShunt have expanded clinical options for glaucoma management, offering distinct advantages in lowering IOP and stabilizing disease progression.

AI and multimodal data integration have accelerated the accuracy of early glaucoma diagnosis, disease progression prediction, and surgical outcome assessments. AI-driven approaches, incorporating genetic and systemic health data, enable more personalized treatment strategies.

As technologies and therapies continue to evolve, clinical practice must emphasize individualized treatment, integrating diagnostic, therapeutic, and prognostic considerations to maximize visual function preservation and quality of life. Future research should prioritize long-term safety, efficacy, and interdisciplinary collaboration while balancing cost-effectiveness and accessibility to establish a truly comprehensive approach to glaucoma care.
